# Primary health care challenges: insights from older people with multimorbidity in Malawi – a qualitative study

**DOI:** 10.1186/s12889-024-18947-3

**Published:** 2024-05-29

**Authors:** Duncan Kwaitana, Maya Jane Bates, Esnath Msowoya, Dorothee van Breevoort, Thomas Mildestvedt, Eivind Meland, Eric Umar

**Affiliations:** 1 Department of Family Medicine, Kamuzu University of Health Sciences, Blantyre, Malawi; 2 Partners in Hope Hospital, Lilongwe, Malawi; 3 Department of Global Public Health and Primary Care, University of Bergen, Oslo, Norway; 4 Department of Health Systems and Policy, Kamuzu University of Health Sciences, Blantyre, Malawi

**Keywords:** Malawi, Older people, Multimorbidity, Low- and middle-income Countries, Access, Primary health care

## Abstract

**Background:**

The global population is undergoing a significant surge in aging leading to increased susceptibility to various forms of progressive illnesses. This phenomenon significantly impacts both individual health and healthcare systems. Low and Middle Income Countries face particular challenges, as their Primary Health Care (PHC) settings often lack the necessary human and material resources to effectively address the escalating healthcare demands of the older people. This study set out to explore the experiences of older people living with progressive multimorbidity in accessing PHC services in Malawi.

**Methods:**

Between July 2022 and January 2023, a total of sixty in-depth interviews were conducted with dyads of individuals aged ≥ 50 years and their caregivers, and twelve healthcare workers in three public hospitals across Malawi’s three administrative regions. The study employed a stratified selection of sites, ensuring representation from rural, peri-urban, and urban settings, allowing for a comprehensive comparison of diverse perspectives. Guided by the Andersen-Newman theoretical framework, the study assessed the barriers, facilitators, and need factors influencing PHC service access and utilization by the older people.

**Results:**

Three themes, consistent across all sites emerged, encompassing barriers, facilitators, and need factors respectively. The themes include: (1) clinic environment: inconvenient clinic setup, reliable PHC services and research on diabetic foods; (2) geographical factors: available means of transportation, bad road conditions, lack of comprehensive PHC services at local health facility and need for community approaches; and (3) social and personal factors: encompassing use of alternative medicine, perceived health care benefit and support with startup capital for small-scale businesses.

**Conclusion:**

This research highlights the impact of various factors on older people’s access to and use of PHC services. A comprehensive understanding of the barriers, facilitators, and specific needs of older people is essential for developing tailored services that effectively address their unique challenges and preferences. The study underscores the necessity of community-based approaches to improve PHC access for this demographic. Engaging multiple stakeholders is important to tackle the diverse challenges, enhance PHC services at all levels, and facilitate access for older people living with progressive multimorbidity.

**Supplementary Information:**

The online version contains supplementary material available at 10.1186/s12889-024-18947-3.

## Introduction

In an era marked by unforeseeable public health hurdles, one undeniable trend emerges—the global population is aging swiftly [1, 2]. Multimorbidity, which refers to the concurrent existence of multiple chronic conditions, becomes more prevalent as individuals age [3]. In the context of research in Africa, the World Health Organization (WHO) classifies older people as individuals aged ≥ 50 years [4].

Worldwide, numerous healthcare systems were originally structured to cater to a relatively youthful population, prioritizing curative care aligned with different health needs than those currently encountered by the older populations [5, 6]. This has created numerous challenges for the older people to easily access Primary Health Care (PHC) services. Primarily, older people in Low and Middle Income Countries (LMICs), such as Malawi, often face challenges in accessing primary healthcare services. This difficulty arises, among others, from the lack of designated clinics specifically catering to their needs [7].

The presence of multimorbidity, compounded by advanced age, leads to significantly increased healthcare utilization and elevated social care costs [8]. Community health care facilities face significant challenges in delivering comprehensive PHC services across many LMICs [5, 9–11]. These challenges stem from the inherent weaknesses in PHC systems, marked by severe shortages in healthcare infrastructure, human resources, as well as essential medicines and supplies [12]. A recent systematic review evaluating the access and utilization of PHC services for older individuals across LMICs identified various additional barriers [9]. These barriers encompassed negative healthcare attitudes, considerable distances to PHC facilities, extended waiting times, insufficient integration of PHC with other services, and instances of stigma and discrimination due to old age and multimorbidity. Consequently, a significant proportion of older people experiencing progressive multimorbidity fail to either access or fully realize the optimum advantages of utilizing PHC services, whether at the community or higher-level facilities. This, in turn, adversely contributes to the deterioration of their health, and in the worst cases, may result in preventable deaths [13].

Malawi, a low-income country in Southern Africa, has a population of approximately 17.6 million, with around 1.6 million being older people (718,479 males and 868,059 females) [14]. There is paucity of evidence regarding experiences of older people living with multimorbidity in accessing PHC services in the Sub-Saharan Africa [15]. Multimorbidity can manifest as various combinations of progressive conditions in older people, such as diabetes, hypertension, cancer, HIV, heart failure, renal failure, arthritis, and various forms of non-communicable diseases (NCDs) [9]. The scarcity of research on the prevalence of multimorbidity in Malawi makes it challenging to accurately assess the national prevalence by age or sex. However, the United Nations (UN) emphasizes the necessity of aligning health and long-term care systems to effectively address the requirements of aging populations [16]. This study aimed to explore experiences of older people with multimorbidity as they seek health care in the Malawi PHC system. The findings are intended to inform policies and practice geared towards enhancing access and healthcare provision for this demographic in a resource poor setting.

## Methods

### Study design

This is a PHC services research that employed a cross sectional qualitative phenomenological design. This approach was chosen in order to elucidate the core of a phenomenon by examining it through the lens of those who undergo it, with the aim of comprehending the significance that participants attribute to the phenomenon [17]. In addition, we used exploratory interviews to facilitate the assessment of the factors affecting access and utilization of PHC services by older people living with progressive multimorbidity. The study was conducted from 18 July 2022 to 02 January 2023.

### General setting: south, centre and north regions of Malawi

Malawi is divided into 28 districts within three major administrative regions, namely: South, Center and North [18]. This study was carried out in three districts; one from each of the three regions and featuring the presence of PHC. In the South, the study site was at Mangochi district hospital, a 500-bed secondary and public health facility. Mangochi is a peri-urban district with a projected 2023 population of 1,346,740 inhabitants [18]. In the Central region, the study site was at Kamuzu Central Hospital, a tertiary government referral facility with a bed capacity of 1,250 and serving approximately 5 million people annually [19]. Kamuzu Central Hospital is in Lilongwe, the capital city of Malawi. Mzimba district hospital (~ 274-bed capacity), another secondary and public health facility, was the third study site located in a rural district of Mzimba in the Northern region of Malawi serving a total population of 940,184 persons [14]. Malawi has four central hospitals, two located in the southern region and one each in the central and northern regions. Additionally, district hospitals are distributed across the country, serving urban, peri-urban, and rural areas. To ensure a representative sample for our study, we employed a stratified sampling approach for study sites, considering various settlement types: rural, peri-urban, and urban. This also allowed for a diverse comparison of experiences of participants in view of PHC services influenced by cultural, socioeconomic and spiritual factors. We excluded mission and private hospitals because of user fees, which may hinder some older people from accessing care. Notably, some LMICs, including Malawi, still primarily deliver PHC services at higher-level facilities such as secondary and tertiary hospitals either due to lack of expertise or resources [9].

In Malawi, patients incur no charges for treatment at public health facilities. These facilities primarily cater to the general population, and there are currently no public or private facilities specifically designated for the older people in the country [7]. There are only a few modest community initiatives aimed at older individuals, and these initiatives are predominantly associated with the activities of civil society or non-governmental organizations [7, 20].

### Participant selection

A purposive sampling frame was used specifically targeting older people living with multimorbidity to explore their experiences regarding access to and utilization of PHC services. The older people also assisted in identifying their caregivers for enrollment in the study. There was a total of 60 adult patients and caregivers (*n* = 20 at Mangochi District Hospital, *n* = 20 at Kamuzu Central Hospital, *n* = 20 at Mzimba District Hospital) who enrolled in this study and participated in in-depth interviews. Additionally, a total of 12 healthcare worker qualitative interviews (*n* = 4 at Mangochi District Hospital, *n* = 4 at Kamuzu Central Hospital, *n* = 4 at Mzimba District Hospital) were conducted across all the three sites. We specifically selected healthcare workers with at least six months of experience in the primary care setting. The selection criteria for all participants were designed to ensure that they possessed sufficient experience from which valuable insights could be gained regarding central issues aligned with the research project’s objectives. Table 1 presents characteristics of study participants.

**Table 1:** Characteristics of older people/caregiver dyads and healthcare workers participating in qualitative interviews at Mangochi and Mzimba district hospitals and Kamuzu central hospital, July 2022 - January 2023

No.	District/Region	Name of Health Facility	Category	Participant Characteristics	*n* = 72 (F = 39)
1	Mzimba - North	Mzimba District Hospital	Rural	Older people with multi-morbidity	10 (6)
				Carer givers of older people	10 (8)
				Healthcare service providers	4 (3)
2	Mangochi - South	Mangochi District Hospital	Peri-urban	Older people with multi-morbidity	10 (9)
				Carer givers of older people	10 (6)
				Healthcare service providers	4 (2)
3	Lilongwe - Centre	Kamuzu Central Hospital	Urban	Older people with multi-morbidity	10 (7)
				Carer givers of older people	10 (8)
				Healthcare service providers	4 (0)

F: Female

### Study theoretical framework

**Fig. 1:**
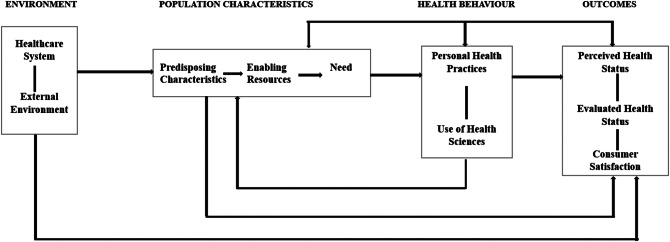
Andersen-Newman Theoretical Framework of Health Services Utilization

Our analysis draws on the Andersen-Newman theoretical framework of health services utilization (Fig. 1) [21]. The framework predicts that a series of factors; predisposing, enabling and need factors influence the utilization of health services by people. In this paper, we focused mainly on employing the four domains of facilitating factors, barriers, need factors and outcomes within the framework. These domains were utilized to examine a range of factors affecting the access and utilization of PHC services by older people living with progressive multimorbidity. While we chose not to extensively analyze the remaining domains within the framework, it is plausible that certain findings could still impact those domains.

### Data collection

Well-trained Research Assistants (RAs) conducted the recruitment processes of study participants. Pre- screening of potential study participants was done during clinic days for the various chronic conditions with assistance from hospital focal point persons. Separate arrangements were made for participants who preferred recruitment from their respective communities.

Exploratory in-depth qualitative interviews were conducted with eligible participants, including older people, caregivers, and healthcare workers, each interviewed individually. The interviews were conducted in any of the following local languages; Chichewa, Yao or Tumbuka including English especially with healthcare workers. The interview guides utilized in this study were specifically tailored for our research and had not been previously published elsewhere (S1 and S2). To ensure rigor in data collection and analysis, all the interviews were digital recorded.

### Data management

Data audio recordings were transcribed verbatim by research assistants. The interviews that were conducted in local languages with older people and caregiver dyads were translated during transcription. Interviews with health care workers were conducted in English, and therefore did not require translating. Unique codes were assigned to each participant for anonymity of data and enhanced ease of identification. All study related tools in hard copies were kept in lockable cabinets for purposes of data safety and only accessible by the researchers. Similarly, a password protected laptop was used to maximize safety of storage of all study data and only the principal investigator had the right of access. Two study team members (DK and EsMs) thoroughly reviewed all transcripts to ensure their completeness.

### Data analysis

The analysis began with data emersion, where two researchers (DK and EsMs), independently read the transcripts multiple times. This process was done inductively with the aim of comprehending the data and pinpoint emerging themes and sub-themes that captured the experiences of study participants in relation to PHC access and utilization. Subsequently, the two researchers convened multiple times to deliberate on the identified themes and sub-themes, reaching a consensus on the development of a code book. The formulation of the code book was guided by the deductive Andersen-Newman thematic framework analytical approach, which emphasizes barriers, facilitators, and need factors related to accessing healthcare services. The transcripts were then imported into NVIVO version 12 pro and systematically coded by DK. The results of the data analysis process were deliberated upon during a study team meeting with the rest of the researchers.

## Results

A total of thirty older person/caregiver dyads (*n* = 60) were recruited from across the three study sites, which were all public health facilities. Individuals in the older age group ranged from 54 to 89 years, whereas caregivers spanned in age from 20 to 82 years. Twelve healthcare professionals, each with a minimum of six months of experience in PHC settings across the study sites, participated in this study.

Our findings unveiled three major themes: (I) clinic environment (II) geographical factors, (III) social factors. These themes functioned as key barriers, facilitators and need factors for accessing PHC services. Below, we elaborate on these thematic areas in the context of facilitators, barriers and need factors, and how they impacted older people in accessing PHC. The corresponding data is depicted in Table 2.

**Table 2:** Thematic areas underpinned by andersen-newman theoretical framework

No.	Major themes	Facilitators	Barriers	Need factors
1	Clinic environment	Reliable PHC services	Inconvenient clinic setup	Research on diabetic foods
2	Geographical factors	Available means of transportation	Bad road conditions	Community approaches
			Lack of comprehensive PHC services at the local facility	
3	Social and personal factors	Perceived healthcare benefit	Use of alternative medicine	Support with startup capital for business

### Facilitators

The Andersen-Newman theoretical framework delineates facilitating factors as elements related to the logistical aspects of accessing healthcare [21]. These factors create conditions that empower an individual to align with a particular value or fulfill a health-related need, influencing their utilization of health services. We utilized this domain of the framework to evaluate the factors driving older people to access PHC services.

### Clinic environment

Participants in the study identified structural clinic environment facilitators as key contributors to the reliability of PHC services, serving as motivation for older individuals to seek and access care.

### Reliable PHC services

Older people expressed that public hospitals were their preferred choice for accessing primary health care services due to the perceived reliability of these services. This observation remained consistent across all study sites, as older people were compelled to bypass their local health centers due to either the unavailability or limited availability of PHC services. Some older people highlighted the consistent availability of medications for their multimorbidity as a compelling factor, serving as a significant motivation for them to consistently choose the higher-level health facilities for their care. Public hospitals were favored primarily due to their non-imposition of user fees. This preference stemmed from the fact that a significant majority of older people lacked a stable source of income to cover associated medical expenses:

*“The quality of care here is good. We don’t pay for any services; we get the services for free.”* (Female, age range 76–80, patient, Mzimba).

Caregivers, often accompanying older individuals to public health facilities, shared similar sentiments. They utilized this experience as a tool to encourage the older people to consistently access (PHC) services.

“……*on that one, it would encourage them because at this hospital, here at the central hospital (Kamuzu Central Hospital), where we are, it is the most reliable hospital in this district and so we still try to come here to receive care*”. (Female, age range 36–40, caregiver, Lilongwe).

### Geographical factors

Despite significant distances, older people were incentivized to access and utilise PHC services due to the presence of transportation assistance.

### Available means of transportation

The considerable distance to PHC facilities posed a significant challenge for older people, making it difficult for them to walk due to physical limitations. In Mangochi and Mzimba sites, some participants travel distances ranging from 10 to 30 km to reach the district hospital. Consequently, any external support towards transportation was greatly appreciated:

*“What I know is that the hospital collaborates with MSF (Médecins Sans Frontières) through a project which economically empowers the patients especially those with cancer of the cervix in terms of transportation to and from the hospital”*. (Palliative care clinic nurse, Mangochi)

However, in Lilongwe, distances are notably shorter, usually less than 10 km, resulting in the absence of external transportation support from organizations for PHC. Similarly, although distances to the district hospital in Mzimba are comparable to those in Mangochi district, no such support was mentioned for Mzimba district either.

### Social and personal factors

Perceived healthcare benefits, served as the primary drivers that encouraged older people to continue accessing PHC.

### Perceived health care benefit

Older people derived extra motivation to access and utilize PHC services, either from personal satisfaction stemming from perceived healthcare benefits or through encouragement from both family members and healthcare workers:

*“No, the hospital is my refuge. It’s my source of assistance, and what they say is accurate. The agony I endured from ulcers, had I opted for traditional medicine, I might not be alive today”*. (Female, age range 56–60, patient, Lilongwe).

### Barriers

While not prominently featured as a primary domain, barriers are acknowledged to coexist with the predisposing factors within the Andersen-Newman theoretical framework [23]. These factors serve as obstacles that hinder the access and utilization of healthcare services, and participants were prompted to elucidate how they were impacted by them.

### Clinic environment

The structural clinic environment barrier, as perceived by participants, primarily revolves around the setup of PHC clinics.

### Inconvenient clinic setup

Older people spent a significant amount of time at the clinics, leading to the need for restroom facilities that presented various challenges. Conversely, older people noted lack of a designated PHC operating space at Mangochi District Hospital. Consequently, they expressed experiencing frequent relocation within the facility, with some of these alternative locations deemed suboptimal for efficient service delivery.

“*The lack of a toilet at our primary care facility has emerged as a significant issue for us”*. (Female, age range 51–55, patient, Mangochi).

On the contrary, primary health care providers across the three sites expressed satisfaction with the clinic setup, primarily attributing it to the favorable location of the PHC clinic. Similar sentiments were echoed by some older people and their caregivers, who also highlighted comparable reasons for their satisfaction.

*“The setting is fine because it is not within where other activities happen, it’s a different place, it’s a different setting. Away from other places so the environment is not stigmatizing”.* (NCD clinic nurse, Mangochi).

### Geographical factors

The accessibility of PHC facilities at secondary or tertiary levels faced challenges, primarily attributed to geographical factors of either bad road conditions or the unavailability of primary care services at the community health centers.

### Bad road conditions

Respondents from Mangochi and Mzimba districts expressed concern with the poor conditions of the roads, compounded by the considerable distance between their communities and the primary care facilities. This resulted in a significantly lengthy travel time, which was not conducive for the older people dealing with the consequences of their multimorbidity throughout the entire journey:

*“The distance that we cover to access the primary health care is too long, the road that we use is not good either”.* (Male, age range 21–25, caregiver, Mangochi).

*“Difficulties arise during the rainy season due to two swampy areas along the route to the hospital, making it very challenging to navigate through”.* (Male, age range 26–30, caregiver, Mzimba).

The roads connecting the villages and communities in Mangochi and Mzimba are unpaved and typically not maintained, exacerbating their condition during the rainy season. However, this concern was not raised by respondents in Lilongwe, where the roads, being in the capital city, are significantly improved, with most of them being tarmac roads.

#### Lack of comprehensive PHC services at the local health facility

Older people reported a deficiency in PHC services at community health centers across all the sites, citing either a lack of skilled healthcare professionals or essential medicines and supplies to address their medical conditions. Consequently, they expressed the necessity to travel extensive distances to access the services at referral hospitals:

*“The sad thing is that from the areas where we come from, clinics are there but when we go there, they always say that they don’t have medicine and test kits for the conditions that we have”.* (Female, age range 51–55, patient, Mangochi).

### Social and personal factors

The interviews revealed a pattern among older individuals who turn to alternative medicine, driven by treatment fatigue at PHC facilities and a belief in its potential to provide healing for their various health conditions.

#### Use of alternative medicine

Various factors were identified as reasons why certain older individuals avoid utilizing PHC services. Among these factors is a preference for relying on herbal medicine and belief in faith healing. This sentiment was common across all study sites:

*“There are many herbal medicines nowadays and other things and so they are told to use such in the hopes that they would get better.”* (NCD clinic nurse, Lilongwe).

*“Some religious beliefs can actually discourage people from going to the hospital to access help. Some even make people to stop taking medications like ARVs (Antiretroviral therapy).”* (Female, age range 56–60, caregiver, Mzimba).

#### Need factors

Need factors within the Andersen-Newman theoretical framework play a crucial role by representing solutions that service users believe can enhance service utilization. Hence, we employed this framework domain to solicit participants’ perspectives on strategies they would suggest to enhance access and utilization of PHC services.

#### Clinic environment

The older people underscored the significance of dedicating resources to research initiatives. Their belief rested on the premise that research endeavors would uncover innovative approaches, ultimately leading to enhancements in their overall quality of life.

### Research on diabetic foods

Older people and their caregivers strongly advocated for increased research on diabetic foods, driven by the hope that a more diverse range of affordable options would become available. This sentiment was rooted in their acute awareness of the economic constraints they faced. Many older people lacked a stable source of income and relied on insufficient handouts, making it challenging to afford the currently available diabetic foods. Consequently, adhering to the recommended diabetic diet seemed like an unattainable goal for them.

*“The researcher should also do research into the types of food that people with diabetes can afford so that we do not end up malnourished”*. (Male, age range 56–60, patient, Lilongwe).

*“We kindly request those responsible to conduct research aimed at identifying affordable and readily accessible dietary options (diabetic diet)”.* (Female, age range 51–55, patient, Mangochi).

### Geographical factors

The absence of PHC services at local health centers posed significant challenges for older people. Their physical conditions often made it impractical for them to walk to secondary or tertiary hospitals to access PHC. Additionally, the financial strain associated with paying for transportation presented a persistent obstacle. In response to these challenges, older people and their caregivers across the three sites recommended the implementation of community-based approaches as a sustainable solution to address this bottleneck.

## Community approaches

Respondents proposed either capacitating local health centers with expert personnel and resources to manage primary care conditions at local level or having scheduled community visits by the hospital primary care teams to attend to patients at the community health facilities:

“*I believe that receiving care and medications at our local health centers would be a highly commendable intervention if it were feasible*”. (Female, age range 56–60, caregiver, Mzimba).

### Social and personal factors

To foster self-reliance and improve their quality of life, older individuals voiced a robust desire for assistance in the form of startup capital for small-scale business ventures.

### Business support

Acknowledging the ongoing economic challenges, older people and their caregivers proposed their inclusion in cash transfer initiatives or similar loan facilities offered by various institutions, or receiving donations to initiate small-scale business ventures. They expressed optimism that such interventions could establish a reliable source of income, consequently ensuring food security at home and alleviating transportation and other related challenges when accessing PHC services at various health facilities:

“*Older people should also get support to have startup capital to run small-scale businesses in order to be self-reliant but also facilitate ease of access and utilization of primary health care services*”. (Female, age 55–60, patient, Mangochi).

## Discussion

The Andersen-Newman theoretical framework of health services utilization was useful in illuminating several factors influencing accessibility of PHC services by older people with progressive multimorbidity in Malawi [21]. Older people may or may not access PHC for a range of reasons, spanning from personal factors to issues within the healthcare system [7].

Our research demonstrated that access to and utilization of primary healthcare services by older people are influenced by a combination of barriers, facilitators, and need factors. These factors can be classified into three overarching thematic categories: clinic environment, geographical factors and social and personal factors. In general, the barriers, facilitators, and need factors exhibited substantial similarity across the three study sites. This likeness can be attributed to the public nature of these facilities, where services were offered free of charge at the point of delivery [22, 23]. Consequently, the shared aspect of providing services without cost likely contributes to comparable experiences across the sites.

The barriers identified in this research align with those identified in a systematic review of previous studies on factors affecting the accessibility of PHC services for older people living with progressive multimorbidity in LMICs [9]. In all three study locations, a recurring observation was the absence of comprehensive PHC services at the community health facilities. This aligns with the results of a previous study conducted in Nigeria [24]. These facilities primarily served as referral points to the next level as they lacked both expert PHC providers and quality essential medicines and supplies [25]. A referral to the higher-level facility (secondary or tertiary) was also met with additional geographical barriers that arose due to poor road conditions for Mangochi and Mzimba sites. This not only made travel uncomfortable for older individuals but also prolonged the journey significantly. Nonetheless, no significant road condition barriers emerged in Lilongwe, owing to its capital status which has spurred substantial enhancements in the road infrastructure, setting it apart from the other two locations. The referral hospital’s inconvenient clinic setup, marked by poorly designed infrastructure, presented an extra barrier for the older people as described elsewhere [3, 26, 27]. In contrast to the two other study sites, older people in Mangochi faced challenges in adapting to the ongoing makeshift administrative arrangements concerning the venues for PHC clinics at the hospital. Due to enduring such exasperating administrative ordeals, some older people turned to alternative medicine, opting for either traditional and herbal remedies or seeking solace in faith healing practices. Moreover, the utilization of alternative medicine in Mzimba and Lilongwe primarily stemmed from personal beliefs rather than frustrations with the PHC clinic. The research substantiates the overarching idea that numerous contextual barriers hinder the access and utilization of primary healthcare services by older people [9]. Older people and their caregivers across the three study sites reported positive experiences with the PHC clinics. They appreciated the convenient location, flexible service provision, and friendly staff. Primary care providers also endorsed the clinic setup, finding it well-suited for delivering PHC services.

The identified facilitating factors align with prior research in resource-limited settings, reflecting a concurrent trend [9]. Nevertheless, our study has unveiled novel insights. Specifically, we found that the perceived benefits of healthcare played a crucial moderating role in shaping individuals’ navigation through various barriers. Notably, satisfaction with advancements in personal health outcomes emerged as a significant motivating factor, driving the ongoing utilization of PHC [28]. The economic hurdles linked to transportation and associated healthcare costs disproportionately affected older people, imposing a substantial burden [10, 11, 29]. Recognizing this issue, certain partner organizations, such as Médecins Sans Frontières (MSF), collaborated with the Ministry of Health to alleviate the challenges faced by older people in Mangochi District. They extended assistance by offering transport support for scheduled monthly clinic visits, thereby enhancing access to healthcare [30]. This support underscores Mangochi District’s capacity to meet a significant demand for PHC for older people with cervical cancer, with a notably higher proportion compared to Mzimba and Lilongwe, where a comparable level of support appears to be absent. Similarly, all the study sites, being public health facilities, did not charge user fees. This healthcare financing mechanism remains applicable to most LMICs following revelations that user fees serve as a barrier to accessing health services [31]. Moreover, our study findings corroborate this, underscoring it as a notable determinant of older peoples’ access to PHC.

Remarkably, older people aged 57 to 88, alongside their caregivers aged 60 to 65, underscored the necessity for support in obtaining startup capital to establish small-scale businesses. This initiative aims to enhance financial independence, consequently facilitating access to and utilization of PHC services. As such, older people aged 57 to 88, in particular, advocated for their inclusion in social cash transfer initiatives, typically overlooked, or sought opportunities to access loan facilities. This proposition aligns with the World Health Organization’s (WHO) report on aging and health, which advocates for the implementation of social assistance measures, including social pensions and related social protection initiatives, in favor of older people [32]. Emphasis should however be placed on the importance of cultivating the capabilities of the older people by allocating resources to their healthcare and long-term support. The objective is to optimize their functional capacity, sustain productive work, and enhance overall health-related quality of life [33].

These findings should be considered within the broader context of general deficiencies observed in PHC settings in Malawi. PHC facilities seemed to be entrenched in inflexible structures, where the understanding, or lack thereof, regarding diverse challenges faced by older people or service delivery had minimal impact on driving enhancements in service delivery. Paradoxically, this is happening despite the Malawian government’s active promotion of the implementation of the National Policy for Older Persons [7]. The policy aims to improve elderly-friendly health services and advocates for its seamless integration into existing health facilities, with the goal of enhancing the management of chronic diseases associated with aging. This is in accordance with the World Health Organization’s emphasis on healthcare centered around individuals [34]. It involves incorporating their perspectives and acknowledging them as both participants and beneficiaries within dependable health systems. In the midst of an economic downturn, it is crucial for the Malawi government to foster increased collaboration with relevant stakeholders. This is necessary to build a robust PHC foundation that can effectively address the diverse care needs of older people living with progressive multimorbidity [12].

The results of this research offer supplementary insights into the state of PHC for older people with multimorbidity in Malawi, carrying implications for policies and practices in LMICs.

### Strengths and limitations

The present study exhibits various strengths, particularly its multi-site approach and the involvement of diverse stakeholders across study locations. This methodology bolsters the study’s robustness by providing a comprehensive contextual understanding of the reported experiences of older individuals living with progressive multimorbidity. However, some older people and their caregivers might have viewed research assistants as integral members of the primary care team. This potential perception could introduce a power imbalance, leading participants to respond differently than they typically would, thereby posing a threat to the internal validity of the data. Nevertheless, the overall consistency in responses helps alleviate this concern.

## Conclusion

The study assessed the barriers, facilitators, and need factors influencing PHC service access by the older people. Access to and utilization of PHC services by older people are influenced by a mix of barriers, facilitators, and need factors, classified into three main categories: clinic environment, geographical factors, and social and personal factors. Provision of community-based comprehensive PHC services can significantly enhance access for older people living with multimorbidity, particularly in resource-constrained settings like Malawi. This approach not only improves the quality of care at the local level but also reduces the necessity for referrals to higher-level facilities, thereby easing the burden on the healthcare system and ensuring timely and efficient care for those in need. A multi-sectoral approach is essential to address infrastructure deficiencies at referral hospitals and improve road conditions, especially in rural areas. This will facilitate easier and more reliable access to referral health facilities when the need arises.

### Electronic supplementary material

Below is the link to the electronic supplementary material.

Supplementary Material 1

Supplementary Material 2

## Data Availability

Data is available on written request from the corresponding author.

## Abbreviations

COMREC: College of Medicine Research and Ethics Committee
LMICs: Low and Middle Income Countries
MSF: Médecins Sans Frontières
NCD: Non-Communicable Diseases
PHC: Primary Health Care
RAs: Research Assistants
UN: United Nations
WHO: World Health Organization
