# Supplementary material for: Primary health care challenges: insights from older people with multimorbidity in Malawi – a qualitative study
Source: BMC Public Health. 2024 May 29;24:1434. doi: 10.1186/s12889-024-18947-3 (PMC11134922; doi:10.1186/s12889-024-18947-3)
Supplement: Supplementary file 2 — Supplementary Material 2 [file 12889_2024_18947_MOESM2_ESM.docx]

**English Interview Guide – Healthcare provider of older people with multimorbidity**

**PARTICIPANT ID: ___________**

**DATE OF INTERVIEW: _____________**

**Part A – INTRODUCTION**

Thank you for agreeing to have this interview with me today. Before we start, I would like to know more about you.

**Part B – DEMOGRAPHIC DATA**

- Occupation ___________
- Gender _________ M/F

**Part C – Perceptions on primary healthcare services for older patients:**

1. What is your general understanding of primary healthcare services?

Probe on:

- Components of primary healthcare services
- Conditions for primary healthcare services
- Frequency of clinic visits

1. Tell me about your experiences providing primary health care services to the older patients?

Probe on:

- Enrollment and initiation to primary care
- Processes of subsequent care
- Availability of medicines and resources
- Explore occurrence of mistreatment and abuse and possible trigger factors
- Variations in treatment of older patients, economic status and other related factors.

1. In your opinion, what do you think about clients waiting time?

Probe on:

- Waiting time upon arrival to the facility/first physical
- What strategies have been put in place to reduce waiting time?

1. What are your experiences regarding communication to older patients and their caregivers?

Probe on:

- Language of communication - How do healthcare workers deal with older patients with language barriers/communication challenges?
- What strategies do midwives use to ensure that older patients and their caregivers have understood the information given?

1. Tell me about the common problems that older patients living with multi-morbidity face when accessing/utilizing primary healthcare services.

Probe on:

- Distance to the healthcare facility
- Availability of resources to access primary healthcare services e.g. income, means of transportation, food and accommodation.
- State of the multimorbid conditions

**Part D – Barriers to access and utilization of primary healthcare services**

1. Explain to me in detail the factors that impede uptake and utilization of primary healthcare services by older patients living with multi-morbidity?

Probe on:

- Shame, guilt.
- Knowledge of primary healthcare services
- Stigma
- Environment/clinic setup
- Quality of primary healthcare services
- Lack of autonomy
- Culture and Family norms
- Distance to health facility
- Health care worker attitude
- Financial/material resources
- Waiting time

**Part E – Enablers/facilitators to primary healthcare services uptake and utilization**

1. Explain to me the factors that enable/facilitate older patients living with multi-morbidity to access and use primary healthcare services

Probe on

- Knowledge
- Environment/clinic setup
- Quality of health services
- Autonomy
- Culture and Family norms
- Distance to health facility
- Health care worker attitude
- Financial/material resources

**Part F – Strategies for improving access and utilization of primary healthcare services by the older patients with multi-morbidity**

1. Explain to me the strategies that can be employed to improve access and utilization of primary healthcare services by the older patients living with progressive multi-morbidity?

Probe on:

- Ask Shifting Approaches
- Patient Centered Approaches
- Community based approaches
- Facility Based approaches

**Part G – Closing Remarks**

We are now at the end of the Interview, Is there anything that you would like to add concerning access and utilization of primary healthcare services by the older patients living with progressive multi-morbidity?

Do you have any question for me?

Thank you very much for your time.
